# Thermal sensitivity of metabolic rate mirrors biogeographic differences between teleosts and elasmobranchs

**DOI:** 10.1038/s41467-023-37637-z

**Published:** 2023-04-12

**Authors:** Yuuki Y. Watanabe, Nicholas L. Payne

**Affiliations:** 1 National Institute of Polar Research, Tachikawa, Tokyo Japan; 2 Department of Polar Science, The Graduate University for Advanced Studies, SOKENDAI, Tachikawa, Tokyo Japan; 3 School of Natural Sciences, Trinity College Dublin, Dublin, Ireland; 4 Present Address: Research Center for Integrative Evolutionary Science, The Graduate University for Advanced Studies, SOKENDAI, Hayama, Kanagawa Japan

**Keywords:** Ecophysiology, Ichthyology

## Abstract

Environmental temperature affects physiological functions, representing a barrier for the range expansions of ectothermic species. To understand the link between thermal physiology and biogeography, a key question is whether among-species thermal sensitivity of metabolic rates is mechanistically constrained or buffered through physiological remodeling over evolutionary time. The former conception, the Universal Temperature Dependence hypothesis, predicts similar among- and within-species thermal sensitivity. The latter conception, the Metabolic Cold Adaptation hypothesis, predicts lower among-species thermal sensitivity than within-species sensitivity. Previous studies that tested these hypotheses for fishes overwhelmingly investigated teleosts with elasmobranchs understudied. Here, we show that among-species thermal sensitivity of resting metabolic rates is lower than within-species sensitivity in teleosts but not in elasmobranchs. Further, species richness declines with latitude more rapidly in elasmobranchs than in teleosts. Metabolic Cold Adaptation exhibited by teleosts might underpin their high diversity at high latitudes, whereas the inflexible thermal sensitivity approximated by Universal Temperature Dependence of elasmobranchs might explain their low diversity at high latitudes.

## Abstract (web summary)

Although diverse species of teleost fishes are present in polar waters, sharks and rays are relatively rare. This study presents analyses to explain this biodiversity pattern, showing that among-species thermal sensitivity of resting metabolic rates is lower than within-species sensitivity in teleosts, but not in sharks and rays.

## Introduction

**Fig. 1:**
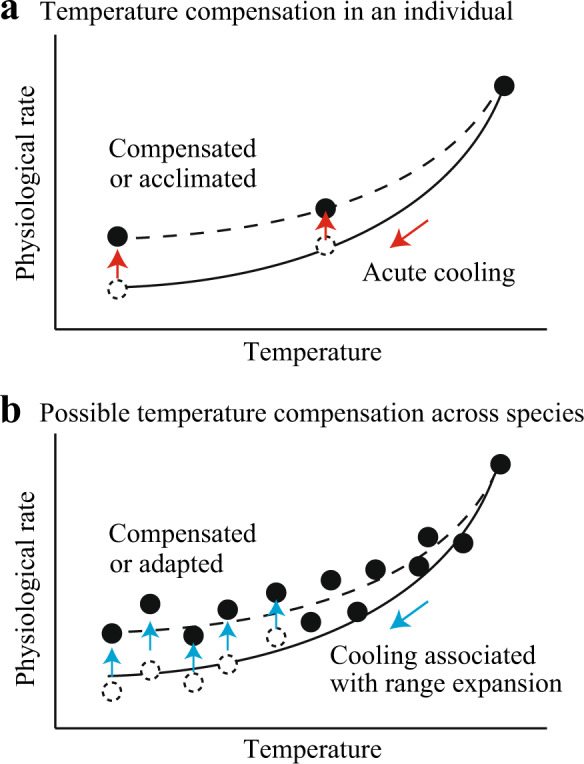
Conceptual graphs showing possible temperature compensation of physiological rate (e.g., metabolic rate) in an individual and among species over evolutionary time. **a** Experimentally induced acute cooling causes a rapid decline in physiological rate of an individual, but the rate can recover to some extents following sufficient time. This process is called acclimation and represents reversible phenotypic changes. **b** Across species, physiological rate tends to decline with decreasing environmental temperature. As such, range expansion into cold waters associated with speciation will cause a decline in physiological rate. However, the effects of temperature may be reduced compared to that predicted from the temperature dependence of individual animals, given that physiological remodeling occurs over evolutionary time. This unreversible temperature compensation could be called adaptation if it occurs.

Environmental temperature is a major barrier for the range expansion of species. For marine ectotherms, range expansion into subpolar and polar regions presents particularly severe challenges, because their physiological functions are impacted by permanently cold waters and the impacts can be lethal to many species^1^. Among many important physiological functions, energy metabolism and its thermal sensitivity are of direct relevance to life history and ecology^2^ and have been extensively studied. Metabolic rates of individual animals, normally measured as oxygen consumption rates of animals in captivity, decline with decreasing temperature. However, ectothermic species have some capacity to reduce the thermal sensitivity of their metabolic rates if they are given sufficient time to remodel their physiology under new thermal regimes (a process called acclimation) (Fig. 1a). Previous experiments showed that Q_10_ [the factorial increase (or decrease) in physiological rates associated with a 10 °C increase (or decrease) in temperature] of metabolic rates of acclimated animals is lower than that of animals exposed to an acute temperature change, especially in aquatic taxa^3^. A key question in an evolutionary context is whether thermal sensitivity of metabolic rates across species can also be reduced by the remodeling of physiology associated with genetic changes in phenotypes (Fig. 1b).

There are two different conceptions. The Universal Temperature Dependence (UTD) hypothesis states that metabolic rates scales with temperature in a similar fashion within and among species because of an inflexible thermal sensitivity of biochemical reactions^4^. The other conception is that thermal sensitivity of metabolic rates across species is more complex and can be adjusted via physiological remodeling and genetic changes over evolutionary time^5^. A form of this latter conception is the Metabolic Cold Adaptation (MCA) hypothesis, which states that the metabolic rate of cold-adapted species is higher at their low temperatures than would be predicted from the observed thermal sensitivity of temperate or tropical species^6,7^. If UTD is correct, the interspecific Q_10_ calculated across species from a range of climate zones would be similar to intraspecific Q_10_ of acclimated, individual species. If MCA is correct, interspecific Q_10_ measured across polar-to-tropical species would be lower than intraspecific Q_10_.

Fishes represent an ideal model to explore thermal sensitivity of metabolic rates and its possible link to biogeography due to their extraordinary high diversity across different climate zones. Diverse fishes are present even in polar waters, and polar fishes are often more active than the warm-water fishes placed in cold waters^8^. Driven by these curious observations, many previous studies tested MCA using various methods and datasets, providing mixed support^6–10^. Crucially, however, these previous tests, and indeed most studies that examined the interspecific relationships of metabolic rates in fishes^11,12^ (including tests of UTD^4,13^), were based overwhelmingly on data for teleosts, with a few elasmobranch species included if any. Bony fishes (Osteichthyes, including teleosts) and cartilaginous fishes (Chondrichthyes, including elasmobranchs) diverged from common ancestors of jawed vertebrates (gnathostomes) about 450 Myr ago^14^. With the long evolutionary separation, teleosts and elasmobranchs have many distinct biological traits, including energy metabolism^15^, but share many environmental and ecological constraints. Curiously, although both groups are distributed globally in today’s oceans, elasmobranchs are relatively rare in the Southern Ocean^16^. Accordingly, comparing thermal sensitivity of metabolic rates and biogeography between teleosts and elasmobranchs could provide clearer evidence for UTD or MCA and insights into the roles of physiological remodeling regarding energy metabolism in the range expansion of species.

In this study, we compile the published reports of resting metabolic rates (RMRs) of thermally acclimated elasmobranchs as widely as possible (i.e., for different species and for different temperatures and body masses within a species). By combining this dataset with previously compiled datasets on teleost RMRs^11,12^, we explore among- and within-species patterns of thermal sensitivity in the two major clades of fishes. Further, we analyze large datasets on marine fish diversity^17,18^ to test whether teleosts and elasmobranchs have expanded toward the poles to similar extents. We hypothesize that the thermal sensitivity of metabolic rates mirrors latitudinal gradient of species richness. That is, a group exhibiting a clearer signature of MCA is predicted to have a higher species diversity at high latitudes than a group to which UTD is better fitted.

## Results

**Fig. 2:**
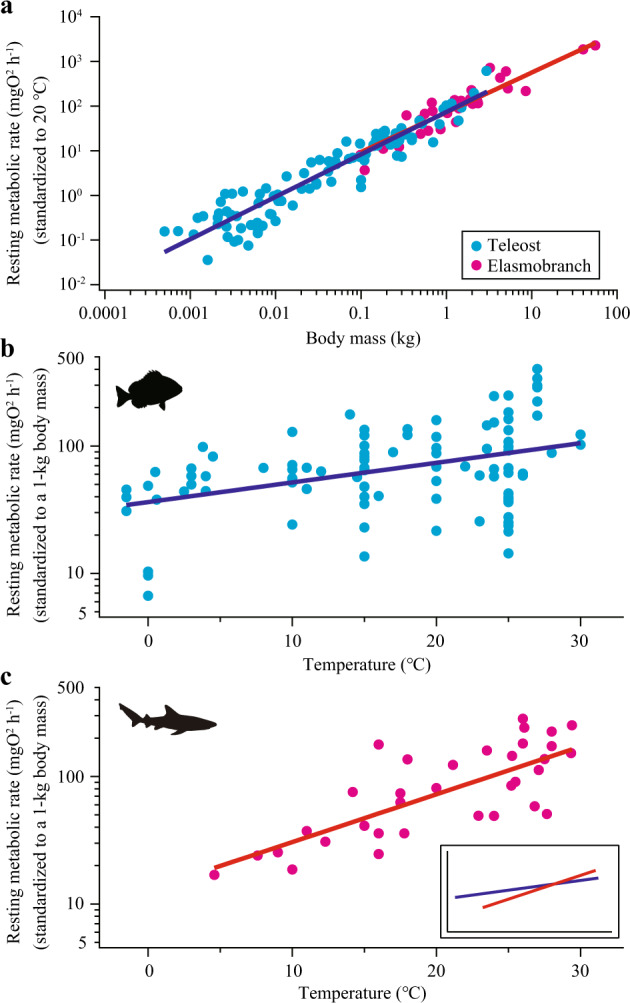
Interspecific allometry and temperature dependence of resting metabolic rates (RMRs) in teleosts (light blue) and elasmobranchs (pink). **a** Allometric relationships of RMRs standardized to 20 °C using the interspecific Q_10_ (1.43 for teleosts and 2.37 for elasmobranchs) estimated with the phylogenetic generalized least squares (PGLS) method. Thick lines are the estimates of RMRs for a temperature of 20 °C based on PGLS [teleost, log_10_(RMR) = 0.95*log_10_(mass) + 1.87; elasmobranch, log_10_(RMR) = 0.89*log_10_(mass) + 1.86]. **b**, **c** Temperature dependance of RMRs standardized to a 1-kg body mass using the allometric slope (0.95 for teleosts and 0.89 for elasmobranchs) estimated with PGLS. Thick lines are the estimates of RMRs for a 1-kg body mass based on PGLS [teleost, log_10_(RMR) = 0.0154*temp + 1.56; elasmobranch, log_10_(RMR) = 0.0375*temp + 1.11]. **c** Inset shows a comparison between teleosts and elasmobranchs. Source data are provided as a Source Data file.

RMRs of elasmobranchs composed of 377 estimates from 34 species (mean body mass 0.10–55.6 kg, temperature 4–31 °C) were compiled (Supplementary Data. 1). A dataset on teleost RMRs previously compiled^12^ was filtered using the same criteria as we used for elasmobranchs (Methods), leaving 100 species (mean body mass 0.0005 − 3.0 kg, temperature −1.5 − 30 °C) (Supplementary Data. 2). Elasmobranchs had larger body mass than teleosts and the mass ranges overlapped only partially. A phylogenetic generalized least squares (PGLS) model with log_10_(RMR) as the response variable and log_10_(mass) and temperature as the predictor variables showed that the allometric slopes (with 95% confidence interval) were similar between teleosts [0.95 (0.88 − 1.02)] and elasmobranchs [0.89 (0.75 − 1.01)] (Fig. 2a). By contrast, temperature dependance was different (Fig. 2b, c). Interspecific Q_10_, reflecting the slope of regression lines in Fig. 2b, c, was lower in teleosts [1.43 (1.14 − 1.76)] than elasmobranchs [2.37 (1.81 − 3.30)] with no overlaps of 95% confidence intervals. This result primarily stemmed from higher teleost RMRs at low temperatures, rather than lower teleost RMRs at high temperature, compared to elasmobranch RMRs (inset in Fig. 2c). Phylogenetic signals, quantified by *d* values that can range from 0 to 1^19^, were 0.28 and 0.13 for teleosts and elasmobranchs, respectively. These *d* values indicate some tendency for closely related species to have similar RMRs for a given body mass and temperature. Model selection analyses using the combined dataset of the two clades (teleost and elasmobranch) showed that the best model for explaining log_10_(RMR) has log_10_(mass), temperature, clade, and the interaction between temperature and clade as predictor variables (Table 1). Thus, the effect of temperature on RMRs across species was clearly different between teleosts and elasmobranchs, whereas that of body mass was not.

**Table 1:** Model selection based on Akaike Information Criterion (AIC) with clade (teleost or elasmobranch) as a categorical predictor variable

Model	AIC	ΔAIC
**log**_**10**_**(RMR)~log**_**10**_**(mass)+temp** + **clade** + temp*clade	**37.33**	0
log_10_(RMR)~log_10_(mass)+temp	38.64	1.31
log_10_(RMR)~log_10_(mass)+temp+clade+log_10_(mass)*clade+temp*clade	38.66	1.33
log_10_(RMR)~log_10_(mass)+temp+clade	40.60	3.27
log_10_(RMR)~log_10_(mass)+temp+clade+log_10_(mass)*clade	41.11	3.78

Regressions were performed with the phylogenetic generalized least squares (PGLS) method. The best model with lowest AIC is denoted by bold.

**Fig. 3:**
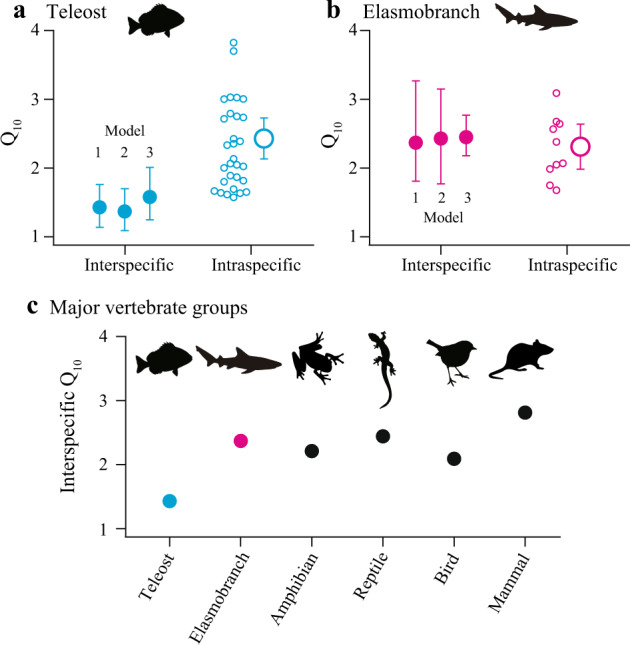
Among- and within-species thermal sensitivity (Q_10_) of resting metabolic rates can differ. **a**, **b** Interspecific Q_10_ (with 95% confidence interval) estimated from the coefficient of the temperature term of three phylogenetic regression models (Models 1–3) compared to intraspecific Q_10_ (shown for each species and as the mean and 95% confidence interval) for **a** teleosts and **b** elasmobranchs. Models 1–3 are a phylogenetic generalized least squares (PGLS) model, a PGLS model with species’ lifestyle as the additional predictor variable, and a phylogenetic mixed model, respectively (Methods). Intraspecific Q_10_ of teleosts is positively skewed, and an outlier (5.4) is not shown. Sample size for teleosts **a**: 100 species for all models of interspecific Q_10_, 31 species for intraspecific Q_10_. Sample size for elasmobranchs **b**: 34 species for Models 1 and 2 and 377 estimates for Model 3 of interspecific Q_10_, 10 species for intraspecific Q_10_. **c** A comparison of interspecific Q_10_ of resting metabolic rates across major vertebrate groups. Data for teleosts and elasmobranchs are from this study and data for other groups are from White et al.^11^.

Interspecific Q_10_ based on the PGLS model (Model 1) was compared to intraspecific Q_10_. In teleosts, interspecific Q_10_ was lower than intraspecific Q_10_ [mean 2.41 (2.10 − 2.71)] (Table S1) estimated for 31 species using a different published dataset^11^, with no overlaps of 95% confidence interval (Fig. 3a). In elasmobranchs, by contrast, interspecific Q_10_ was similar to intraspecific Q_10_ [mean 2.29 (1.96−2.61)] (Table S1) estimated for 10 species using our dataset (Fig. 3b). These results did not change (i) when the variation of lifestyles (i.e., pelagic, benthopelagic, or demersal) among species (Table S2) was added as a categorical predictor variable (Model 2), or (ii) when multiple measures from single species of elasmobranchs, rather than the average of each species, were included in a phylogenetic mixed model (Model 3) (Fig. 3a, b). Moreover, stricter rules for data inclusion were applied and only data for thermally acclimated individuals for ≥2 weeks prior to measurements were analyzed. Despite decreased sample sizes (from 100 to 50 species in teleosts and from 34 to 19 species in elasmobranchs), the subset still showed a large difference in interspecific Q_10_ between teleosts [1.41 (1.07−1.74)] and elasmobranchs [2.17 (1.42−3.31)] based on PGLS. Comparing our results with previous estimate of interspecific Q_10_ of RMRs for major vertebrate groups^11^, interspecific Q_10_ of elasmobranchs was not exceptionally high. Rather, interspecific Q_10_ of teleosts was exceptionally low (Fig. 3c).

**Fig. 4:**
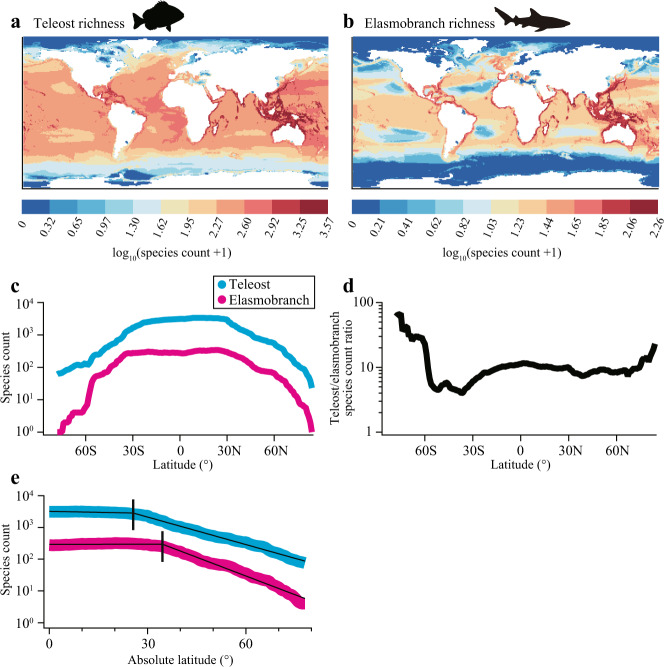
Contrasting diversity of marine teleosts and elasmobranchs at high latitudes. **a**, **b** Diversity maps created by AquaMaps for **a** teleosts and **b** elasmobranchs, with areas with relatively high and low diversity shown in red and blue, respectively. Elasmobranch diversity is disproportionally low at high latitudes, especially in the Southern Ocean. **c** Species count in a log scale plotted against latitude for teleosts (light blue) and elasmobranchs (pink) based on FishBase. **d** Teleost/elasmobranch species count ratio (in a log scale) plotted against latitudes, showing that it rises at high latitudes, especially in the southern hemisphere. **e** Species count (in a log scale) plotted against absolute latitude for teleosts and elasmobranchs, fitted with the broken-line regression model. At latitudes above the breakpoints (vertical bars), species count declines with latitude more rapidly in elasmobranchs than teleosts. Source data for **c**–**e** are provided as a Source Data file.

Based on AquaMaps^17^, species richness declines at high latitude in both teleosts and elasmobranchs. However, the richness of elasmobranchs at high latitudes is disproportionally low, especially in the southern hemisphere (Fig. 4a, b). Our investigation of FishBase^18^ showed that teleosts are approximately 10 times more speciose than elasmobranchs for a given latitude lower than 60°. The teleost/elasmobranch ratio of species count rose at higher latitudes, especially in the southern hemisphere, reaching 68 at 75°S (Fig. 4c, d). Species count averaged across the northern and southern hemisphere for a given absolute latitude showed that it is rather stable at low and middle latitudes but declines with latitude at higher latitudes (Fig. 4e). The broken-line regression analyses^20^ indicated that the breakpoint latitude is 25.6 and 34.5° for teleosts and elasmobranchs, respectively. Beyond the breakpoint latitudes, the log_10_ of species count declined with latitude more rapidly for elasmobranchs [0.0394 degree^−1^ (0.0379 − 0.0408)] than teleost [0.0289 degree^−1^ (0.0283 − 0.0295)], with no overlaps of 95% confidence interval.

## Discussion

### Contrasting thermal sensitivity of metabolic rates

We found that the among-species influence of temperature on RMRs is strikingly different between teleosts and elasmobranchs. By contrast, the among-species influence of body mass is similar and individual species from both clades have similar intraspecific Q_10_ (Figs. 2 and 3). Our estimate of interspecific Q_10_ of elasmobranch RMRs (2.37) is consistent with the previous study that analyzed a smaller dataset (2.23)^21^. The interspecific Q_10_ of teleost RMRs (1.43) is somewhat different from the previous study that shared data with this study but had less strict rules for data inclusion (1.62)^12^ and an older study that used a different dataset and statistical method (1.83)^7^. Due to this variability, a range of 1.4 − 1.8 can be considered the best estimate for interspecific Q_10_ of teleost RMRs. In all such case, teleosts have lower interspecific Q_10_ than elasmobranchs, although no previous studies compiled data for both clades and compared them with a consistent methodology. Lower interspecific Q_10_ of teleosts arises primarily from elevated RMRs of cold-water species, despite large variability for RMR at a given temperature in this clade (Fig. 2b, c). Notably, interspecific Q_10_ is lower than intraspecific Q_10_ in teleosts (as shown before^7^), whereas interspecific and intraspecific Q_10_ of RMRs are similar in elasmobranchs. This finding is robust, as shown by the three different phylogenetic regression models we tested (Fig. 3a, b). Interspecific Q_10_ of teleost RMRs is remarkably low even among major vertebrate groups (Fig. 3c). Taken together, our results present strong support for MCA in teleosts but not in elasmobranchs. RMRs of elasmobranchs are better explained by UTD that assumes an inflexible thermal sensitivity among and within species.

Metabolic rates are an integrative proxy for a variety of fitness-related processes and traits, including enzyme reactions, locomotion, growth, and reproduction^22^. Besides RMRs we analyzed in this study, other metrics of metabolic rates, such as maximum metabolic rates and aerobic scope (i.e., the difference between maximum and resting metabolic rates), are ecologically relevant^23^; however, these metrics are positively associated with one another across species at least in teleosts^12^. Therefore, our finding of reduced thermal sensitivity of RMRs across teleost species suggests that teleosts in cold waters may have better competitive ability than predicted for that temperature at the cost of higher energy expenditure. By contrast, elasmobranch species in cold waters do not appear to exhibit such evolutionary changes as teleosts. This observation is exemplified by Greenland sharks (the species with the lowest water temperature in our elasmobranch dataset) that exhibit unusually slow swim speed^24^, low metabolic rate^21^, and slow growth rate^25^.

We acknowledge that our elasmobranch dataset is still limited compared to teleosts, for which large amounts of data on metabolic rates have long been collected and complied. The data on elasmobranchs should be expanded in future studies with regard to the number of species, phylogeny, lifestyle, temperature coverage, and the types of metabolic rate metrics. Moreover, to better characterize the difference in thermal sensitivity of physiological rates between teleosts and elasmobranchs, other important temperature-dependent traits (e.g., enzyme activity, locomotor performance) of elasmobranchs should be examined in future studies, as previously demonstrated for teleosts^9^.

### Link between metabolic rates and biogeography

Our findings of contrasting thermal sensitivity of RMRs between teleosts and elasmobranchs apparently mirror the contrasting biogeographic patterns of the two groups. Species richness are highest at low and middle latitudes in both teleosts and elasmobranchs, as previously reported^26,27^. However, we found that the rate of declines in species richness with increasing latitudes differs, leading to increased teleost/elasmobranch richness ratio at high latitudes (Fig. 4). This pattern is especially evident in the Southern Ocean, where diverse teleosts are present whereas elasmobranchs are rare^16^. According to FishBase, 247 species (1.5%) of marine teleosts (total, 16,611 species), comprised of 38 families across 12 orders, occur in polar waters. By contrast, only 8 species (0.7%) of marine elasmobranchs (total, 1,198 species), comprised of two genera of skates (*Bathyraja* and *Amblyraja* spp.), are categorized as polar species. Thus, with regard to both the number of extant species and taxonomic diversity, elasmobranchs are less successful in expanding their ranges into cold waters than teleosts. This difference cannot be explained by the development of anti-freeze proteins, which prevent ice formation and growth and are found in the plasma or epidermis (e.g., skin) of multiple linages of polar teleosts^28^. Unlike teleosts, elasmobranchs maintain osmotic equilibrium with the surrounding seawater primarily by high concentrations of urea^29^ and have no risk of freezing even at sub-zero water temperature. Therefore, we propose that MCA exhibited by teleosts underpins their high diversity at high latitudes, whereas the inflexible thermal sensitivity of RMRs approximated by UTD of elasmobranchs is associated with their low diversity at high latitudes.

It is important to note that any ecological benefits associated with MCA (i.e., elevated RMRs in cold waters) come with challenges such as elevated energy requirements. In the environment where resources (e.g., food, oxygen) are scarce, less active lifestyles with reduced RMRs might be favored. For example, some teleosts (e.g., carp) with low RMRs are highly tolerant to hypoxia^12^, allowing them to expand their range into the environment where oxygen concentrations occasionally drop^30^. Tolerance to hypoxia is unlikely an important factor affecting the survivals of marine fishes at high latitudes due to increased dissolved oxygen concentrations in colder waters. However, tolerance to food shortage associated with low RMRs can undoubtedly be a selective advantage for marine fishes at high latitudes, where seasonal fluctuations in food availability is pronounced. As such, while it is possible that the capacity for MCA in teleosts (but not elasmobranchs) facilitated their range expansion into cold waters, causality may be the reverse. Highly successful diversification and range expansions of teleosts may have caused high variability in RMRs for a given temperature (Fig. 2b), which can be interpreted as the evidence of MCA. High variability in teleost RMRs reflects a broad range of ecological niches occupied by this group. Notably, some teleosts (e.g., salmons) have highly active lifestyles in cold waters, despite their ectothermic physiology like many other species. As an approximation, we refer to waters of <15 °C as “cold waters”, because 15 °C lies roughly halfway between typical average sea surface temperature of ~30 °C in the tropics and ~0 °C in polar waters. Teleosts and elasmobranchs have diverged RMRs below 15 °C (Fig. 2b, c). It is our view that, in general, cold-water elasmobranchs are either sluggish (e.g., Greenland sharks^24^) or active with regional endothermic physiology (e.g., salmon sharks^31^). There seem to be no “salmon-like” elasmobranchs, being highly active cold-water ectotherms—a view that can partly explain low elasmobranch diversity at high latitudes. Thus, while stopping short of claiming that one pattern causes the other, we propose that contrasting thermal sensitivities of RMRs between teleosts and elasmobranchs are intrinsically linked to their biogeographic differences.

An unresolved issue is why teleost/elasmobranch richness ratio is much higher at high latitudes in the southern hemisphere (the Southern Ocean) than northern hemisphere (the Arctic Ocean) (Fig. 4). Compared to the Arctic Ocean, the Southern Ocean has an older history of the present thermal regime and higher degree of isolation from adjacent seas^32^. These differences may have caused increased difference in species richness between teleosts (that have capacity for MCA) and elasmobranchs (that do not) in the southern hemisphere compared to the northern hemisphere. Detailed analyses separating northern and southern fauna with taxonomically and geographically expanded datasets on metabolic rates could address this issue in future studies.

### Possible physiological mechanism

An intriguing question arising from our findings is why teleosts have capacity for MCA but elasmobranchs do not. We refer to a previous review article^15^ for the possible physiological mechanism, which will be summarized below.

Teleosts and elasmobranchs are distinct in their pathways of energy metabolism. Adipose tissues are stored as oxidative fuels in teleost muscles but are absent in elasmobranch muscles, where ketone bodies and amino acids are main oxidative fuels^15^. This unusual energy metabolism of elasmobranchs is likely related to their osmoregulatory strategy. Unlike teleosts, elasmobranchs are iso-osmotic to the surrounding seawater primarily by maintaining high concentration of urea^29^. They use amino acids for both osmoregulation (as essential nitrogen donor for urea synthesis) and energy metabolism (as oxidative fuels and ketogenic precursors). Importantly, fatty acids in adipose tissues are preferred oxidative fuels in cold-water teleost species^32^, which often have increased mitochondria density in muscles^33^. Therefore, the inability of elasmobranchs to utilize fatty acids as energy source may limit their RMRs in cold water (as previously suggested^15^), potentially leading to the lack of capacity for MCA.

We acknowledge that our discussion is rather speculative. To advance our understanding, the energy metabolism of cold-water elasmobranchs needs to be studied at lower levels of biological organization (the molecular, organellar, cellular, and tissue levels) than the whole-organism level, as previous studies exclusively targeted cold-water teleosts^32,33^.

In conclusion, we show that the thermal sensitivity of metabolic rates is different between teleosts and elasmobranchs and propose that the difference may underpin their contrasting diversity at high latitudes. In teleosts, among-species thermal sensitivity is lower than within-species thermal sensitivity, consistent with the MCA hypothesis that might explain high diversity of this group at high latitudes. In elasmobranchs, by contrast, among- and within-species thermal sensitivity is similar, consistent with the UTD hypothesis that may be associated with poor diversity of this group at high latitudes. Fishes have been a central study model to explore whether thermal sensitivity of organisms’ metabolic rates are constrained mechanistically by biochemical kinetics^4^, or if it represents a complex interplay between physics and evolutionary adaptations to particular temperature and lifestyle^5^. Findings from this study provide improved precision of our understanding of thermal sensitivities across organisms. It is teleosts – not fishes in general^9^ – that exhibit a clear signature of evolutionary adaptation and depart from the traditional view that interspecific Q_10_ of RMR in vertebrates is approximately 2–3 (Fig. 3C). Understanding the underlying mechanisms of the divergence between teleosts and other vertebrates could help reveal why metabolic rate scales with temperature in the first place and predict how different species will react to changing climates.

## Methods

### Metabolic rate data compilation

Data on the resting metabolic rates (RMRs) of elasmobranchs, measured as the oxygen consumption rates of fasted, thermally acclimated animals during resting (i.e., not actively swimming) periods, were compiled with body mass and water temperature from the literature (Supplementary Data. 1). RMR is an approximation of standard (or basal) metabolic rate in fishes, although the effects of some body movements are often included. In most of the compiled studies, individuals were acclimated to the experimental temperature for ≥1 week and fasted for ≥2 days prior to measurements; however, these conditions were not met in some cases, such as experiments on large species in remote areas^21^. Thermal acclimations of RMR can require days to weeks, depending on the extent and direction of the temperature change^34^. To examine the robustness of our results, the studies that acclimated individual fish for ≥2 weeks were noted and analyzed separately. When RMRs were measured at different controlled temperatures, we ensured that (i) target temperature was achieved slowly (1 °C day^−1^ or less in most cases) and (ii) test temperatures fall within the natural range of the species. When the effects of acute temperature changes on RMRs were reported, we only extracted the estimates for the initial, control conditions. Following a review article^34^, we did not accept RMRs measured for chemically sedated animals, or surgically operated animals (e.g., canulation) that likely incur increased stress. In addition to direct measurements, we accepted the RMRs of continuous swimmers estimated by extrapolating the relationship between metabolic rate and swim speed (or other activity measures such as body acceleration) to zero activity level. The four species for which the extrapolation method was used (i.e., blacknose sharks, blacktip sharks, bull sharks, and scalloped hammerhead sharks) had relatively high RMRs for a given body mass and temperature, likely reflecting their active lifestyles. Limited data available for the species with regional endothermy (e.g., shortfin mako sharks) were excluded in this study. With large body mass and elevated metabolic rates, these species would have disproportionally large effects on allometric relationships, precluding a fair comparison of RMRs between teleosts and elasmobranchs. While some literature only reported the mean RMR of multiple individuals, other literature reported more detailed information (RMR of individual animals and for different temperature treatments) in various forms. We extracted as detailed information as possible by digitalizing the figures or referring to the supplemental materials and data repository of the literature, when applicable. As such, each datapoint in Supplementary Data. 1 represents either an individual or the mean of multiple individuals. Scientific names of species were matched to those in FishBase^18^.

For teleosts, a published dataset of RMRs composed of 112 species^12^, measured within the natural temperature range of the species, was used to examine among-species patterns. We referred to the original literature cited by the dataset and excluded species that do not meet the selection criteria we used for elasmobranchs, leaving 100 species (Supplementary Data. 2). In some instances where reported values did not match our calculation based on the original sources, we used the values we calculated. As for elasmobranchs, the studies with thermal acclimation durations of ≥2 weeks were noted and analyzed separately. We also modified scientific names of species following “The Fish Tree of Life”^26^, a large dataset of bony fish phylogeny that is mostly consistent with the information in FishBase^18^. Because Killen et al.’s dataset^12^ only included species-averaged data, we used a different dataset^11^ to examine intraspecific Q_10_ of teleost RMRs.

### Metabolic rate analyses

RMR data for elasmobranchs were averaged for each species. Log_10_ values, rather than raw values, were averaged for RMR and body mass, because these are the input of subsequent analyses. Possible effects of local adaptations among populations of a species^35^ were considered to be beyond the scope of this study. The phylogenetic generalized least squares (PGLS) method was used to examine the effect of body mass and temperature on RMR across species with the effect of phylogeny accounted for. Phylogenetic trees were created for the compiled species of teleosts and elasmobranchs with the published relationships among species^26,36,37^ and an arbitrary branch length^38^ (Figs. S1 and S2). PGLS analyses were performed with log_10_(RMR) as the response variable and log_10_(mass) and temperature as the predictor variables under the Ornstein-Uhlenbeck evolutionary process model using the Regressionv2 program^19^ of the software Matlab (MathWorks). Interspecific Q_10_ was calculated as 10^10*a^, where *a* is the coefficient (or slope) for temperature in the PGLS analyses. The 95% confidence interval of each estimate was computed by the bootstrap method^19^. Phylogenetic signals were quantified as *d* value (ranging from 0 to 1) using restricted maximum likelihood^19^. *d* = 0 indicates that non-phylogenetic (i.e., the ordinary least squares) model best fit the data, whereas *d* = 1 indicates that the statistical model with the provided branch lengths best fit the data.

To test whether the effects of body mass and temperature on RMRs across species are different between teleosts and elasmobranchs, RMR data for the two groups were combined, and their phylogenetic trees were connected at the roots. Model selection analyses were performed using PGLS with clade (teleost or elasmobranch) as a categorical predictor variable. Five representative models, including those having interaction between clade and other predictor variables, were compared based on Akaike Information Criterion (AIC) (Table 1).

Intraspecific Q_10_ was estimated for 31 species of teleosts (using White et al.’s dataset^11^) and 10 species of elasmobranchs (using our dataset; Supplementary Data. 1), for which RMR data are available over a ≥ 7 °C temperature difference (Table S1). The ordinary least squares regressions were performed with log_10_(RMR) as the response variable and log_10_(mass) and temperature as the predictor variables. As for interspecific Q_10_, intraspecific Q_10_ was calculated as 10^10*a^, where *a* is the coefficient (or slope) for temperature.

In addition to the main PGLS analysis (Model 1), two different models (Models 2 and 3) were tested to examine the robustness in our estimates of interspecific Q_10_. Model 2 also used the PGLS method but had the lifestyle of each species as the additional predictor variable [log_10_(RMR) ~ log_10_(mass) + temp + lifestyle]. We tested this model because metabolic rates are often affected by lifestyles (i.e., species with active lifestyles tend to have elevated metabolic rates)^12^. For each species of our datasets, the categorical information on lifestyle (i.e., pelagic, benthopelagic, or demersal) was extracted from FishBase^18^ with the software R and the package rfishbase^39^ (Table S2). For simplicity, the category of “reef associated” was merged into “benthopelagic”. Model 3 was a phylogenetic mixed model [log_10_(RMR) ~ log_10_(mass) + temp] with phylogeny included as a random factor and was fitted to all datapoints for elasmobranchs (rather than the data averaged for each species). This analysis was performed with the software R and the package MCMCglmm^40^. Model 3 used much larger datasets than Models 1 and 2 for elasmobranchs, making 95% confidence interval narrower (Fig. 3b). The same model was applied for the teleost dataset, which is the data averaged for each species.

### Biogeography analyses

Global maps for the species diversity of teleosts and elasmobranchs were created using AquaMaps^17^ (Fig. 4a, b). The group categories of “bony fish” and “sharks and rays” in AquaMaps were considered nearly the same as teleosts and elasmobranchs, respectively. Latitudinal gradients of species richness were analyzed based on FishBase^18^ with the software R and the package rfishbase^39^. For all existing taxonomic orders of teleosts (following “The Fish Tree of Life”^26^) and elasmobranchs, lists of species were extracted with latitudinal range, habitat types (freshwater, brackish water, and saltwater), and climate zones (e.g., tropical, temperate, boreal, polar). The species that do not occur in saltwater were excluded, leaving 16,611 and 1198 species of marine teleosts and elasmobranchs, respectively. The number of species that occurs for each degree of latitude was calculated both for teleosts and elasmobranchs (Fig. 4c, d). Further, species count was averaged across the northern and southern hemisphere for a given absolute latitude up to 78° (Fig. 4e). The relationships between log_10_(species count) and absolute latitude were examined by the broken-line regression models^20^ with the software R.

### Reporting summary

Further information on research design is available in the Nature Portfolio Reporting Summary linked to this article.

## Supplementary information

Supplementary Information

Description of Additional Supplementary Files

Supplementary Data 1

Supplementary Data 2

Reporting Summary

## Data Availability

Metabolic rate data used in this study are available in Supplementary Data 1 and 2 and the supplementary material of ref. ^11^. Data used in biogeography analyses are available at open databases (AquaMaps and FishBase). Source data are provided with this paper.

## Source data

Source Data

## Code availability

R codes used in this study are available at Github repository (https://github.com/yuuki412/fish_metabolic_rate_and_biogeography).
