# Author Correction: Thermal sensitivity of metabolic rate mirrors biogeographic differences between teleosts and elasmobranchs

**DOI:** 10.1038/s41467-023-38166-5

**Published:** 2023-04-25

**Authors:** Yuuki Y. Watanabe, Nicholas L. Payne

**Affiliations:** 1 National Institute of Polar Research, Tachikawa, Tokyo Japan; 2 Department of Polar Science, The Graduate University for Advanced Studies, SOKENDAI, Tachikawa, Tokyo Japan; 3 School of Natural Sciences, Trinity College Dublin, Dublin, Ireland; 4 Present Address: Research Center for Integrative Evolutionary Science, The Graduate University for Advanced Studies, SOKENDAI, Hayama, Kanagawa Japan

**Keywords:** Ecophysiology, Ichthyology

Correction to: *Nature Communications* 10.1038/s41467-023-37637-z, published online 12 April 2023

In this article the grant number 18/SIRG/5549 relating to Science Foundation Ireland for Nicholas L. Payne was omitted.

The original article has been corrected.

