# Supplementary material for: Thermal sensitivity of metabolic rate mirrors biogeographic differences between teleosts and elasmobranchs
Source: Nat Commun. 2023 Apr 12;14:2054. doi: 10.1038/s41467-023-37637-z (PMC10097821; doi:10.1038/s41467-023-37637-z)
Supplement: Supplementary file 1 — Supplementary Information [file 41467_2023_37637_MOESM1_ESM.pdf]

Supplementary Information for Watanabe and Payne (2023)

This file includes:

Table S1. Intraspecific Q_{10} of resting metabolic rate
Table S2. Lifestyle of elasmobranchs and teleosts
Fig. S1. Phylogenetic tree of elasmobranchs
Fig. S2. Phylogenetic tree of teleosts

Table S1. Intraspecific Q_{10} of resting metabolic rate for elasmobranchs and teleosts.

Species	Temperature range (°C)	Q_{10}
Elasmobranch		
California horn shark *Heterodontus francisci*	14−22	2.07
Clearnose skate *Rostroraja eglanteria*	20−28	1.68
Common eagle ray *Myliobatis aquila*	10−25	1.99
Lemon shark *Negaprion brevirostris*	22−30.7	1.75
Lesser sandshark *Acroteriobatus annulatus*	15−25	2.05
Nurse shark *Ginglymostoma cirratum*	23−30	2.64
Port Jackson shark *Heterodontus portusjacksoni*	16−23.6	2.68
Round stingray *Urobatis halleri*	15−27	3.09
Thorny skate *Amblyraja radiata*	5−13	2.57
Whitespotted bamboo shark *Chiloscyllium plagiosum*	15−30	2.38
Teleost		
Ameiurus nebulosus	10−30	2.39
Anguilla japonica	13−30	3.02
Catostomus commersonii	10−20	2.72
Chaenocephalus aceratus	2−10	1.64
Cirrhinus cirrhosus	20−30.5	3.00
Cyprinus carpio	10−35	1.64
Exodon paradoxus	20−30	3.03
Gadus morhua	5−15	1.80
Gambusia affinis	20−30	1.67
Lampetra fluviatilis	4.4−16	3.70
Lampetra planeri	5.3−15.6	3.82

Limanda limanda	5−15	2.79
Macrognathus aculeatus	21−30	1.61
Micropterus salmoides	15−30	1.82
Microstomus kitt	5−15	1.69
Mugil cephalus	14.5−28.5	2.43
Myxine glutinosa	7−15	1.57
Oncorhynchus mykiss	5−26	2.02
Oncorhynchus nerka	5−25	2.06
Orthodon microlepidotus	5−30	2.13
Petromyzon marinus	5−20	3.00
Platichthys flesus	5−15	2.35
Platichthys stellatus	9−20	2.75
Pleuronectes platessa	5−20	1.87
Salmo salar	6−18	2.33
Pseudopleuronectes americanus	5−24	5.40
Salvelinus fontinalis	5−20	2.05
Salvelinus namaycush	9.1−22.1	2.74
Scyliorhinus canicula	7−17	2.00
Sebastes diploproa	10−20	1.65
Thymallus arcticus	4−12	1.89

Table S2. Lifestyle of elasmobranchs and teleosts used in the analysis (Model 2 in Fig. 3a, b). Species are listed in the same order as phylogenetic trees (Figs. S1, S2).

Species no.	Species	Lifestyle
	Elasmobranch	
1	Greenland shark<br>*Somniosus microcephalus*	benthopelagic
2	Pacific sleeper shark<br>*Somniosus pacificus*	benthopelagic
3	Spiny dogfish<br>*Squalus acanthias*	benthopelagic
4	California horn shark<br>*Heterodontus francisci*	demersal
5	Port Jackson shark<br>*Heterodontus portusjacksoni*	demersal
6	Whitespotted bamboo shark<br>*Chiloscyllium plagiosum*	benthopelagic
7	Epaulette shark<br>*Hemiscyllium ocellatum*	benthopelagic
8	Nurse shark<br>*Ginglymostoma cirratum*	benthopelagic

9	Lesser spotted dogfish<br>*Scyliorhinus canicula*	demersal
10	Swell shark<br>*Cephaloscyllium ventriosum*	demersal
11	Draughtsboard shark<br>*Cephaloscyllium isabella*	benthopelagic
12	Leopard shark<br>*Triakis semifasciata*	demersal
13	Gummy shark<br>*Mustelus antarcticus*	demersal
14	Scalloped hammerheard shark<br>*Sphyrna lewini*	pelagic
15	Bonnethead shark<br>*Sphyrna tiburo*	benthopelagic
16	Blacktip shark<br>*Carcharhinus limbatus*	benthopelagic
17	Whitetip reef shark<br>*Triaenodon obesus*	benthopelagic
18	Blacktip reef shark<br>*Carcharhinus melanopterus*	benthopelagic
19	Lemon shark<br>*Negaprion brevirostris*	benthopelagic
20	Bull shark<br>*Carcharhinus leucas*	benthopelagic
21	Blacknose shark<br>*Carcharhinus acronotus*	benthopelagic
22	Little skate<br>*Leucoraja erinacea*	demersal
23	Thorny skate<br>*Amblyraja radiata*	demersal
24	Clearnose skate<br>*Rostroraja eglanteria*	demersal
25	Marbled electric ray<br>*Torpedo marmorata*	benthopelagic
26	Lesser sandshark<br>*Acroteriobatus annulatus*	demersal
27	Largetooth sawfish<br>*Pristis pristis*	demersal
28	Bat ray<br>*Myliobatis californica*	demersal
29	Common eagle ray<br>*Myliobatis aquila*	benthopelagic
30	Cownose ray<br>*Rhinoptera bonasus*	benthopelagic
31	Atlantic stingray<br>*Hypanus sabinus*	demersal
32	Brown stingray<br>*Bathytoshia lata*	demersal
33	Ribbontail stingray<br>*Taeniura lymma*	benthopelagic
34	Round stingray<br>*Urobatis halleri*	demersal

	Teleost	
1	*Anguilla rostrata*	demersal
2	*Anguilla anguilla*	demersal
3	*Anguilla japonica*	demersal
4	*Anguilla australis*	benthopelagic
5	*Rhinichthys osculus*	demersal
6	*Phoxinus phoxinus*	demersal
7	*Abbottina rivularis*	benthopelagic
8	*Pseudorasbora parva*	benthopelagic
9	*Coreius guichenoti*	benthopelagic
10	*Rhodeus ocellatus*	benthopelagic
11	*Hypophthalmichthys nobilis*	benthopelagic
12	*Hypophthalmichthys molitrix*	benthopelagic
13	*Mylopharyngodon piceus*	demersal
14	*Ctenopharyngodon idella*	benthopelagic
15	*Opsariichthys bidens*	benthopelagic
16	*Zacco platypus*	benthopelagic
17	*Schizothorax wangchiachii*	benthopelagic
18	*Onychostoma simum*	benthopelagic
19	*Spinibarbus sinensis*	benthopelagic
20	*Procypris rabaudi*	benthopelagic
21	*Carassius auratus*	benthopelagic
22	*Rhodeus sinensis*	benthopelagic
23	*Cyprinus carpio*	benthopelagic
24	*Epalzeorhynchos frenatum*	benthopelagic
25	*Labeo rohita*	benthopelagic
26	*Cirrhinus mrigala*	demersal
27	*Danio rerio*	benthopelagic
28	*Ictalurus punctatus*	demersal
29	*Oncorhynchus mykiss*	benthopelagic
30	*Oncorhynchus nerka*	pelagic
31	*Salmo trutta*	pelagic
32	*Salvelinus alpinus*	benthopelagic
33	*Salvelinus fontinalis*	benthopelagic
34	*Coregonus albula*	benthopelagic
35	*Mallotus villosus*	pelagic

36	*Gadus macrocephalus*	demersal
37	*Gadus ogac*	demersal
38	*Gadus morhua*	benthopelagic
39	*Boreogadus saida*	demersal
40	*Merlangius merlangus*	benthopelagic
41	*Melanogrammus aeglefinus*	demersal
42	*Pollachius virens*	demersal
43	*Eleginus gracilis*	demersal
44	*Encheliophis homei*	benthopelagic
45	*Sparus aurata*	demersal
46	*Chrysophrys auratus*	benthopelagic
47	*Scolopsis bilineata*	benthopelagic
48	*Dicentrarchus labrax*	demersal
49	*Argyrosomus japonicus*	benthopelagic
50	*Micropogonias undulatus*	demersal
51	*Leiostomus xanthurus*	demersal
52	*Ammodytes tobianus*	demersal
53	*Myoxocephalus scorpius*	demersal
54	*Myoxocephalus octodecemspinosus*	demersal
55	*Artediellus atlanticus*	demersal
56	*Agonus cataphractus*	demersal
57	*Cyclopterus lumpus*	benthopelagic
58	*Lycodes eudipleurostictus*	demersal
59	*Lycodes pallidus*	demersal
60	*Zoarces americanus*	demersal
61	*Anarhichas lupus*	demersal
62	*Lumpenus lampretaeformis*	demersal
63	*Notothenia neglecta*	benthopelagic
64	*Notothenia rossii*	demersal
65	*Lepidonotothen nudifrons*	demersal
66	*Trematomus hansoni*	demersal
67	*Trematomus pennellii*	demersal
68	*Trematomus nicolai*	demersal
69	*Trematomus bernacchii*	demersal
70	*Pagothenia borchgrevinki*	pelagic
71	*Sander lucioperca*	pelagic

72	*Sander vitreus*	benthopelagic
73	*Lepomis gibbosus*	benthopelagic
74	*Micropterus salmoides*	benthopelagic
75	*Paracirrhites forsteri*	benthopelagic
76	*Paracirrhites arcatus*	benthopelagic
77	*Neocirrhites armatus*	benthopelagic
78	*Kuhlia sandvicensis*	benthopelagic
79	*Typhlogobius californiensis*	demersal
80	*Ostorhinchus cyanosoma*	benthopelagic
81	*Ostorhinchus doederleini*	benthopelagic
82	*Scomber japonicus*	pelagic
83	*Scomber scombrus*	pelagic
84	*Platichthys flesus*	demersal
85	*Pleuronectes platessa*	demersal
86	*Limanda limanda*	demersal
87	*Microstomus kitt*	demersal
88	*Seriola lalandi*	benthopelagic
89	*Aphanius dispar*	demersal
90	*Oreochromis niloticus*	benthopelagic
91	*Forsterygion lapillum*	benthopelagic
92	*Pomacentrus moluccensis*	benthopelagic
93	*Acanthochromis polyacanthus*	benthopelagic
94	*Chromis atripectoralis*	benthopelagic
95	*Dascyllus aruanus*	benthopelagic
96	*Chelon labrosus*	demersal
97	*Liza aurata*	pelagic
98	*Rhinomugil corsula*	pelagic
99	*Mugil curema*	benthopelagic
100	*Mugil cephalus*	benthopelagic

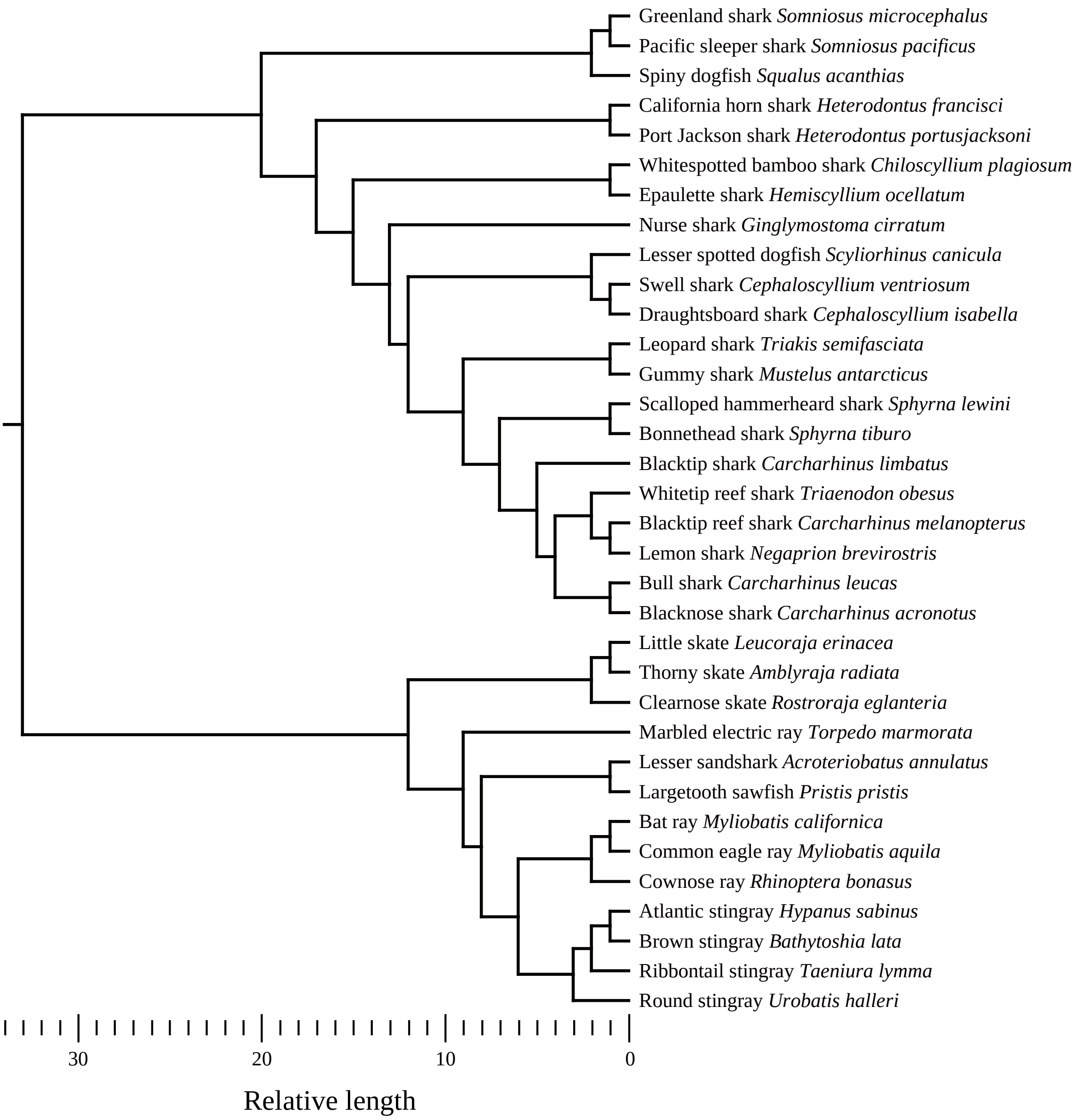

Fig. S1. Phylogenetic tree of elasmobranchs used in the analyses.

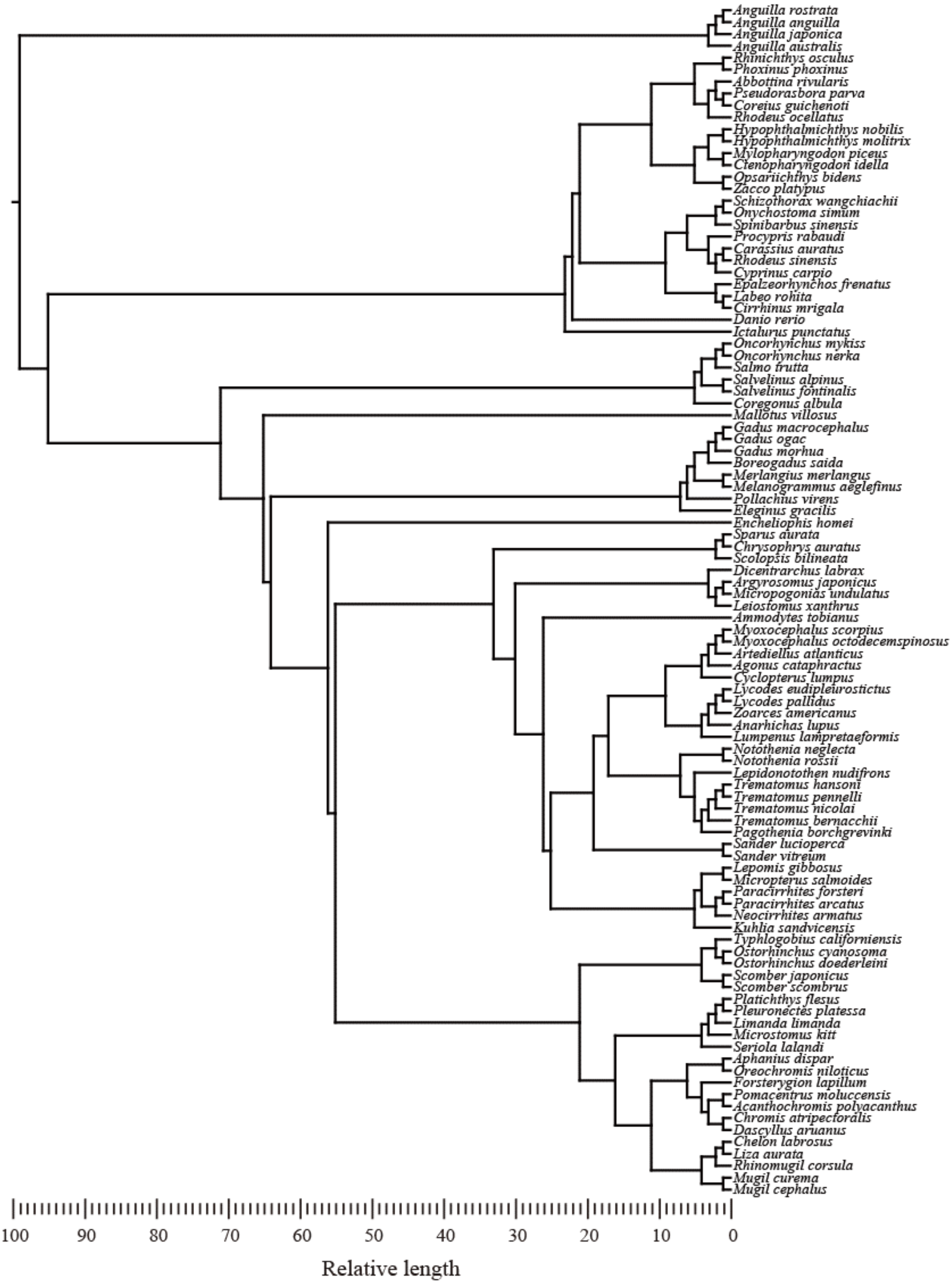

Fig. S2. Phylogenetic tree of teleosts used in the analyses.
