# Supplementary material for: Thermal sensitivity of metabolic rate mirrors biogeographic differences between teleosts and elasmobranchs
Source: Nat Commun. 2023 Apr 12;14:2054. doi: 10.1038/s41467-023-37637-z (PMC10097821; doi:10.1038/s41467-023-37637-z)
Supplement: Supplementary file 2 — Description of Additional Supplementary Files [file 41467_2023_37637_MOESM2_ESM.pdf]

Description of Additional Supplementary Files

File Name: Supplementary Data 1
Description: Resting metabolic rate of elasmobranchs. Species are listed in the same order as phylogenetic tree (Fig. S1).

File Name: Supplementary Data 2
Description: Resting metabolic rate of teleosts. Data compiled by Killen et al. (Ref. 1) were filtered using our selection criteria (Methods) and further checked by referring to the original studies. Scientific names of species were matched to those in “The Fish Tree of Life” (Ref. 2). Information modified from Killen et al’s dataset are denoted in red. Species are listed in the same order as phylogenetic tree (Fig. S2).
