# Supplementary material for: Thermal sensitivity of metabolic rate mirrors biogeographic differences between teleosts and elasmobranchs
Source: Nat Commun. 2023 Apr 12;14:2054. doi: 10.1038/s41467-023-37637-z (PMC10097821; doi:10.1038/s41467-023-37637-z)
Supplement: Supplementary file 5 — Reporting Summary [file 41467_2023_37637_MOESM5_ESM.pdf]

nature portfolio

Corresponding author(s): Yuuki Watanabe

Last updated by author(s): Mar 2, 2023

Reporting Summary

Nature Portfolio wishes to improve the reproducibility of the work that we publish. This form provides structure for consistency and transparency in reporting. For further information on Nature Portfolio policies, see our Editorial Policies and the Editorial Policy Checklist.

Statistics

For all statistical analyses, confirm that the following items are present in the figure legend, table legend, main text, or Methods section.

n/a	Confirmed	
[ ]	[x]	The exact sample size (*n*) for each experimental group/condition, given as a discrete number and unit of measurement
[ ]	[x]	A statement on whether measurements were taken from distinct samples or whether the same sample was measured repeatedly
[x]	[ ]	The statistical test(s) used AND whether they are one- or two-sided<br>*Only common tests should be described solely by name; describe more complex techniques in the Methods section.*
[ ]	[x]	A description of all covariates tested
[x]	[ ]	A description of any assumptions or corrections, such as tests of normality and adjustment for multiple comparisons
[ ]	[x]	A full description of the statistical parameters including central tendency (e.g. means) or other basic estimates (e.g. regression coefficient) AND variation (e.g. standard deviation) or associated estimates of uncertainty (e.g. confidence intervals)
[x]	[ ]	For null hypothesis testing, the test statistic (e.g. *F*, *t*, *r*) with confidence intervals, effect sizes, degrees of freedom and *P* value noted<br>*Give P values as exact values whenever suitable.*
[x]	[ ]	For Bayesian analysis, information on the choice of priors and Markov chain Monte Carlo settings
[x]	[ ]	For hierarchical and complex designs, identification of the appropriate level for tests and full reporting of outcomes
[x]	[ ]	Estimates of effect sizes (e.g. Cohen's *d*, Pearson's *r*), indicating how they were calculated

Our web collection on statistics for biologists contains articles on many of the points above.

Software and code

Policy information about availability of computer code

Data collection	The software R (v 4.1.2) and the rfishbase (v 4.0.0) package were used to extract data from FishBase.
Data analysis	Statistical analyses were carried out in Matlab R2011b with the regressionv2 package and in R (v 4.1.2) with the MCMCglmm (v 2.34) and segmented (v 1.5) packages. R codes used in this study are available at Github repository (https://github.com/yuuki412/fish_metabolic_rate_and_biogeography).

For manuscripts utilizing custom algorithms or software that are central to the research but not yet described in published literature, software must be made available to editors and reviewers. We strongly encourage code deposition in a community repository (e.g. GitHub). See the Nature Portfolio guidelines for submitting code & software for further information.

Data

Policy information about availability of data

All manuscripts must include a data availability statement. This statement should provide the following information, where applicable:

- Accession codes, unique identifiers, or web links for publicly available datasets
- A description of any restrictions on data availability
- For clinical datasets or third party data, please ensure that the statement adheres to our policy

Metabolic rate data used in this study are available in Supplementary Data 1 and 2. Data on biogeography were collected from open databases (AquaMaps and FishBase).

Human research participants

Policy information about studies involving human research participants and Sex and Gender in Research.

Reporting on sex and gender	NA
Population characteristics	NA
Recruitment	NA
Ethics oversight	NA

Note that full information on the approval of the study protocol must also be provided in the manuscript.

Field-specific reporting

Please select the one below that is the best fit for your research. If you are not sure, read the appropriate sections before making your selection.

☐ Life sciences ☐ Behavioural & social sciences ☒ Ecological, evolutionary & environmental sciences

For a reference copy of the document with all sections, see nature.com/documents/nr-reporting-summary-flat.pdf

Ecological, evolutionary & environmental sciences study design

All studies must disclose on these points even when the disclosure is negative.

Study description	Comparative analyses of resting metabolic rates and biogeography in teleosts and elasmobranchs. All data used in this study were collected from the literature and open databases.
Research sample	No research samples were collected in this study.
Sampling strategy	No sampling was conducted in this study.
Data collection	The leading author (Y. Y. Watanabe) collected data from the literature and open databases.
Timing and spatial scale	Data were collected from September 2021 to October 2022.
Data exclusions	Data on metabolic rates were excluded when the experimental protocols described in the papers did not meet our selection criteria.
Reproducibility	Three phylogenetic regression models were tested to confirm that results are consistent.
Randomization	Because no experiments were performed in this study, randomization of test subjects does not apply.
Blinding	Each species in the datasets of metabolic rates was assigned a unique code and the subsequent regression analyses were performed without knowing which species is represented by which data point. This process helped us to avoid any unconscious bias toward potential outcomes.

Did the study involve field work? ☐ Yes ☒ No

Reporting for specific materials, systems and methods

We require information from authors about some types of materials, experimental systems and methods used in many studies. Here, indicate whether each material, system or method listed is relevant to your study. If you are not sure if a list item applies to your research, read the appropriate section before selecting a response.

Materials & experimental systems

n/a	Involved in the study
☒	☐ Antibodies
☒	☐ Eukaryotic cell lines
☒	☐ Palaeontology and archaeology
☒	☐ Animals and other organisms
☒	☐ Clinical data
☒	☐ Dual use research of concern

Methods

n/a	Involved in the study
☒	☐ ChIP-seq
☒	☐ Flow cytometry
☒	☐ MRI-based neuroimaging
